# Supplementary material for: Oxytocin promotes epicardial cell activation and heart regeneration after cardiac injury
Source: Front Cell Dev Biol. 2022 Sep 30;10:985298. doi: 10.3389/fcell.2022.985298 (PMC9561106; doi:10.3389/fcell.2022.985298)
Supplement: Supplementary file 3 [file DataSheet1.DOCX]

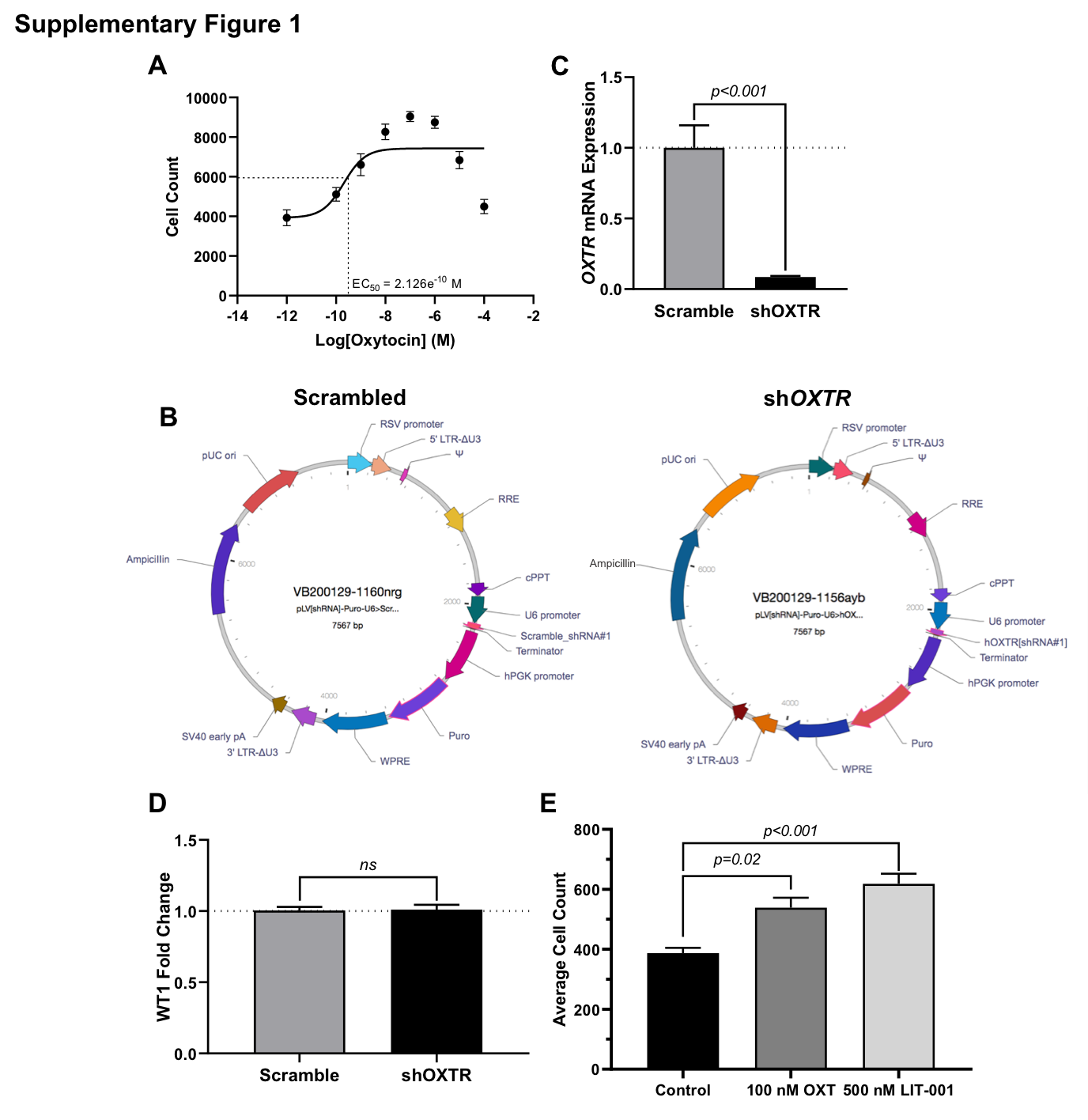


**Supplementary Figure 1**. **A)** Dose-response data for hEpiCs exposed to different concentrations of OXT over the course of 5 days, expressed as number of nuclei at each concentration; n=10 per concentration. **B)** Plasmid used for shRNA-mediated knockdown of *OXTR* in hiPSCs and the corresponding scrambled plasmid. **C)** qRT-PCR for “Scramble” and “shOXTR” hEpiCs after *OXTR* knockdown; n=6 per cell line. **D)** hEpiC differentiation efficiency in both cell lines, expressed as percent WT1+ nuclei relative to each other at day 25 of differentiation; n=20 per cell line. **E)** Absolute cell counts of scramble hEpiCs exposed to 100 nM OXT and 500 nM LIT-001, a non-peptide OXTR agonist; n=12 per condition.


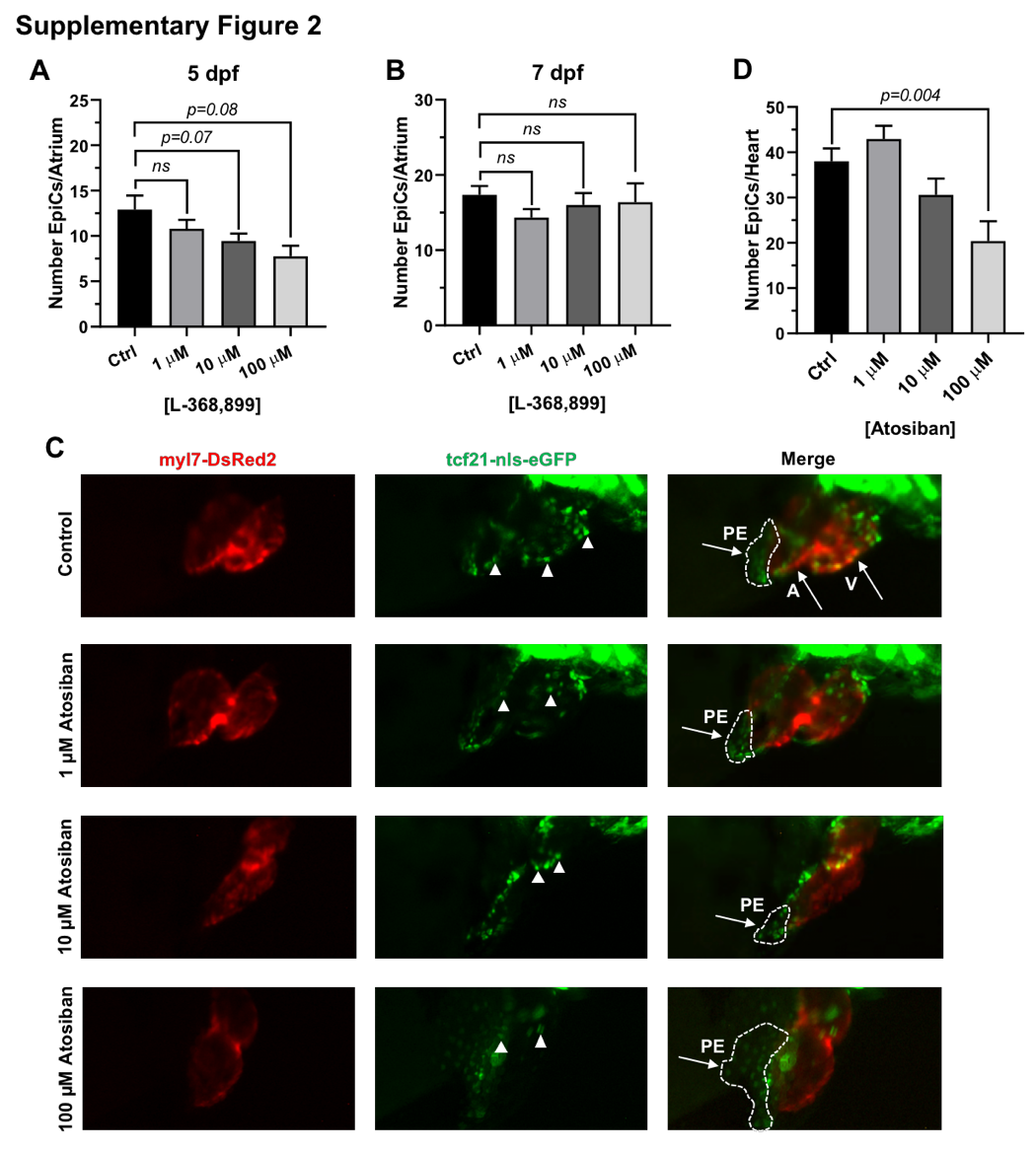


**Supplementary Figure 2**. **A-B)** Epicardial cell counts per atrium of developing zebrafish embryos at 5 and 7 dpf treated with different concentrations of L-368,899. **C-D)** Fluorescent images (C) and epicardial cell counts per heart (D) of developing zebrafish embryos at 3 dpf treated with different concentrations of atosiban. In (C), proepicardial and epicardial cells are labeled with GFP (green dots, arrowheads), myocardium is labeled with DsRed2 (red), dashed lines demarcate proepicardial organ; n≥8 embryos per condition; A: Atrium, PE: Proepicardium, V: Ventricle.


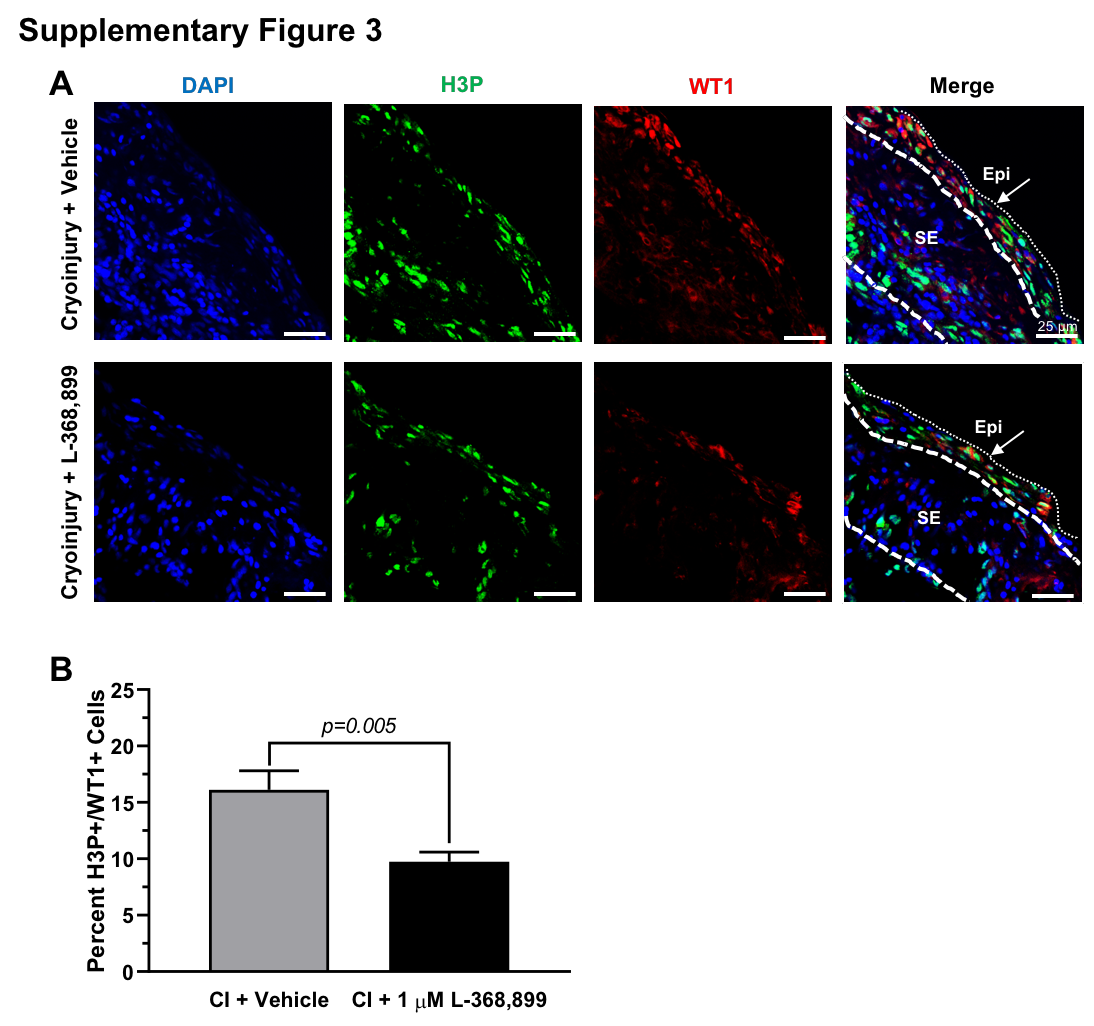


**Supplementary Figure 3**. **A-B)** Confocal immunofluorescent images (A) and quantification (B) of proliferating epicardial cells in cryoinjured zebrafish hearts 3 days after cardiac cryoinjury. In (A), epicardial cells are labeled with WT1 (red), proliferating cells are labeled with H3P (green), nuclei are labeled with DAPI (blue); n=8 images per condition, scale bar: 25 µm; Epi: Epicardium, SE: Subepicardium.
